# Time to remission in childhood steroid sensitive nephrotic syndrome: a change in perspective

**DOI:** 10.1007/s00431-025-06090-z

**Published:** 2025-03-20

**Authors:** Beatrice Nardini, Claudio La Scola, Ciro Corrado, Alberto Edefonti, Mario Giordano, Roberto Pillon, Antonio P. Mastrangelo, Marco Materassi, Irene Alberici, William Morello, Giuseppe Puccio, Giovanni Montini, Andrea Pasini

**Affiliations:** 1 Specialty School of Pediatrics, Alma Mater Studiorum, University of Bologna, Bologna, Italy; 2 Pediatric Nephrology and Dialysis Unit, IRCCS Azienda Ospedaliero-Universitaria Di Bologna, Bologna, Italy; 3 Pediatric Nephrology Unit, Children’s Hospital “G. Di Cristina”, A.R.N.A.S. “Civico”, Palermo, Italy; 4 Pediatric Nephrology, Dialysis and Transplant Unit, Fondazione IRCCS Ca’ Granda Ospedale Maggiore Policlinico, Milan, Italy; 5 Nephrology Unit, XXIII Children’s Hospital, Bari, Giovanni Italy; 6 Florence, Italy; 7 Palermo, Italy; 8 Giuliana and Bernardo Caprotti Chair of Pediatrics, Department of Clinical Sciences and Community Health, University of Milano, Milan, Italy

**Keywords:** Childhood nephrotic syndrome, Time to remission, Steroid therapy, Hypovolemia

## Abstract

Time to remission (TTR) has been largely considered one of the predictive factors for the risk of relapse and steroid dependency in childhood steroid-sensitive nephrotic syndrome, yet conflicting opinions exist. However, the factors influencing TTR have never been studied. We performed a post-hoc analysis of the prospective pediatric cohort enrolled in a previous multicenter study (ClinicalTrials.gov Id: NCT01386957) to evaluate the possible influence of some clinical and laboratory parameters at INS onset on the timing of TTR. A total of 136 children were evaluated. In simple linear regression models, TTR was directly correlated with serum uric acid, urea, potassium, and urinary protein levels at onset. TTR showed a non-linear inverse correlation with age at onset. A multiple linear regression model of TTR showed that hyperuricemia (p = 0.0000007), non linear age (*p* = 0.0006) and proteinuria (especially in binary form) (*p* = 0.02) were significant predictors of TTR, and that there was a significant positive interaction between uricemia and proteinuria as predictors of TTR (*p* = 0.004). *Conclusions*: In our analysis, TTR appears to be associated to a nephrotic status at clinical diagnosis characterized by more severe proteinuria and hyperuricemia. Moreover, younger age at onset, notably associated with prognosis, is also associated with a longer TTR.

**Table:** 

**What is Known:** *• Corticosteroids are the first-line treatment in childhood nephrotic syndrome.* *• Over the years, time to remission has been considered a potential predictive factor for the risk of relapse and steroid dependency in childhood nephrotic syndrome, with conflicting results.*	
**What is New:** *• Clinical and laboratory parameters at nephrotic syndrome onset are associated with prolonged time to remission in children.*

**What is Known:**

*• Corticosteroids are the first-line treatment in childhood nephrotic syndrome.*

*• Over the years, time to remission has been considered a potential predictive factor for the risk of relapse and steroid dependency in childhood nephrotic syndrome, with conflicting results.*

**What is New:**

*• Clinical and laboratory parameters at nephrotic syndrome onset are associated with prolonged time to remission in children.*

## Background

Idiopathic Nephrotic Syndrome (INS) is the most prevalent glomerular disease in children [1]. Corticosteroids are the first-line treatment and induce remission in about 80–90% of patients [2–4]. Time to remission (TTR) is defined as the time, measured in days, from the start of steroid-therapy to the first day of negative or trace dipstick. Over the years, TTR has been considered one of the potential predictive factors for the risk of relapse and steroid dependency. Several authors have identified TTR as a prognostic factor [5–8]. Yap et al. [6] reported that the risk of steroid-dependency was directly related to TTR. Vivarelli et al. [9] defined TTR as an early prognostic factor in INS, with all patients with a TTR > 14 days relapsing within 3 months and approximately 50% of patients with TTR < 7 days still being in remission three months after steroid discontinuation. Prasun et al. [8] observed that TTR had a significant relationship with the pattern of relapse, with infrequent relapses more common in patients that responded in < 7 days, and frequent relapses in patients with a longer TTR. Conversely, other authors have not found any correlation between TTR and disease outcomes [10].

In 2021, we published a prospective, observational, multicenter study [11] designed to categorize patients into two groups, according to TTR (≤ 10 or > 10 days), receiving different steroid regimens to mitigate the supposed higher risk of relapse in late responders. The results of the study did not show a significant role for TTR as a prognostic factor, signaling no indication for a variation of steroid dose based on this parameter.

In addition, to our knowledge, the mechanisms behind the longer TTR observed in some patients with INS have never been studied. For this reason, we decided to perform a post-hoc analysis of the prospective pediatric cohort enrolled in the previous study to evaluate the possible influence of some clinical and laboratory parameters at INS onset on the timing of TTR.

## Methods

We performed a post-hoc analysis of the prospective cohort enrolled in the multicenter study (ClinicalTrials.gov Id: NCT01386957; Registered 30/06/2011) which involved 49 Italian pediatric units. Patients with a first episode of INS, defined as proteinuria > 40 mg/m^2^/h or urine protein/creatinine ratio (uPr/uCr) > 2 mg/mg and albuminemia ≤ 2.5 g/dL and an age at onset between 6 months and 18 years were enrolled. Patients with congenital and secondary forms of nephrotic syndrome or steroid resistance were excluded. From the start of steroid-therapy, dipstick urinalysis was performed daily to precisely identify TTR. Clinical (age, height, weight, body mass index, systolic and diastolic blood pressure (SDS)) and laboratory parameters (complete blood count, urea, creatinine, uric acid, serum protein, albumin, total cholesterol, triglycerides, electrolytes, urinalysis, uPr/uCr) were recorded in an online database (www.nefrokid.it) at diagnosis, 12, and 24 months. Further details on the treatment can be found in Pasini et al. [11]. In our post hoc analysis, we decided to exclude patients who achieved remission after 28 days or more, defined as late responders [3], due to supposed additional mechanisms involved in these patients’ longer response.

The protocol was approved by the Ethics Committee CE-AVEC Emilia-Romagna, Italy (reference number 7/2011/0/Oss) and by the institutional review board of each participating center. Informed consent was obtained from all participants/legal guardians by the treating physicians. The study was conducted in accordance with the Declaration of Helsinki.

### Statistical analysis

All statistical analyses were performed using the open-source software R. The Chi-Square test of independence and Fisher’s exact test were used to analyze the relationship between categorical variables. Non-parametric tests (Wilcoxon, Kruskal–Wallis) were used to analyze the difference in the distribution of a continuous variable in two or more different groups. Simple and multiple linear regression models were used to evaluate the relationship between continuous and categorical predictors and a continuous outcome. Proteinuria was considered both in continuous and in binary form (categorized at a threshold of 10 mg/mg, which was the median value in our sample).

## Results

### Population

A total of 136 children were evaluated, 47 (35%) females and 89 (65%) males. The median age at diagnosis was 3.7 years (range 1.1–15.1). The median TTR was 8 (range 1–25) days. The other variables at onset are described in Table 1.

**Table 1:** Population characteristics at onset

Variable at onset	Median (range)*
Clinical data	
Age, years	3.65 (1.1–15.1)
Sex, %Male	89 (65%)
Height, SDS	−0.02 (−2–2)
Weight, SDS	0.39 (−2–3)
BM, SDS	0.7(−1.8–2.9)
sBP, SDS	0.9(−1.3–3.7)
dBP, SDS	1.2 (−0.7–3.9)
TTR, days	8 (1–25)
Laboratory data	
Hemoglobin (g/dL)	13.2 (9.4–17.6)
Urea (mg/dL)	27 (7–94)
Creatinine (mg/dl)	0.28 (0.1–0.89)
Uric acid (mg/dL)	4.1 (2.4–6.6)
GFR	165 (57–459)
Total protein (g/dL)	4.1 (3.1–6.2)
Albumin (g/dL)	1.6 (0.5–2.5)
Tot. cholesterol (mg/dL)	390 (137–659)
Triglycerides (mg/dL)	175 (50–605)
Na (mmol/L)	137 (126–149)
K (mmol/L)	4.5 (3.5–5.5)
Ca (mg/dL)	8.2 (5–9.4)
P (mg/dL)	5 (3–7.6)
uPr/uCr (mg/mg)	10 (0.9–49)
Urine Output (ml/kg/h)	1.36 (0.17–4.3)

*Median (range) for numerical variables, number (%) for categorical variables

### Clinical and laboratory variables at onset

**Fig. 1:**
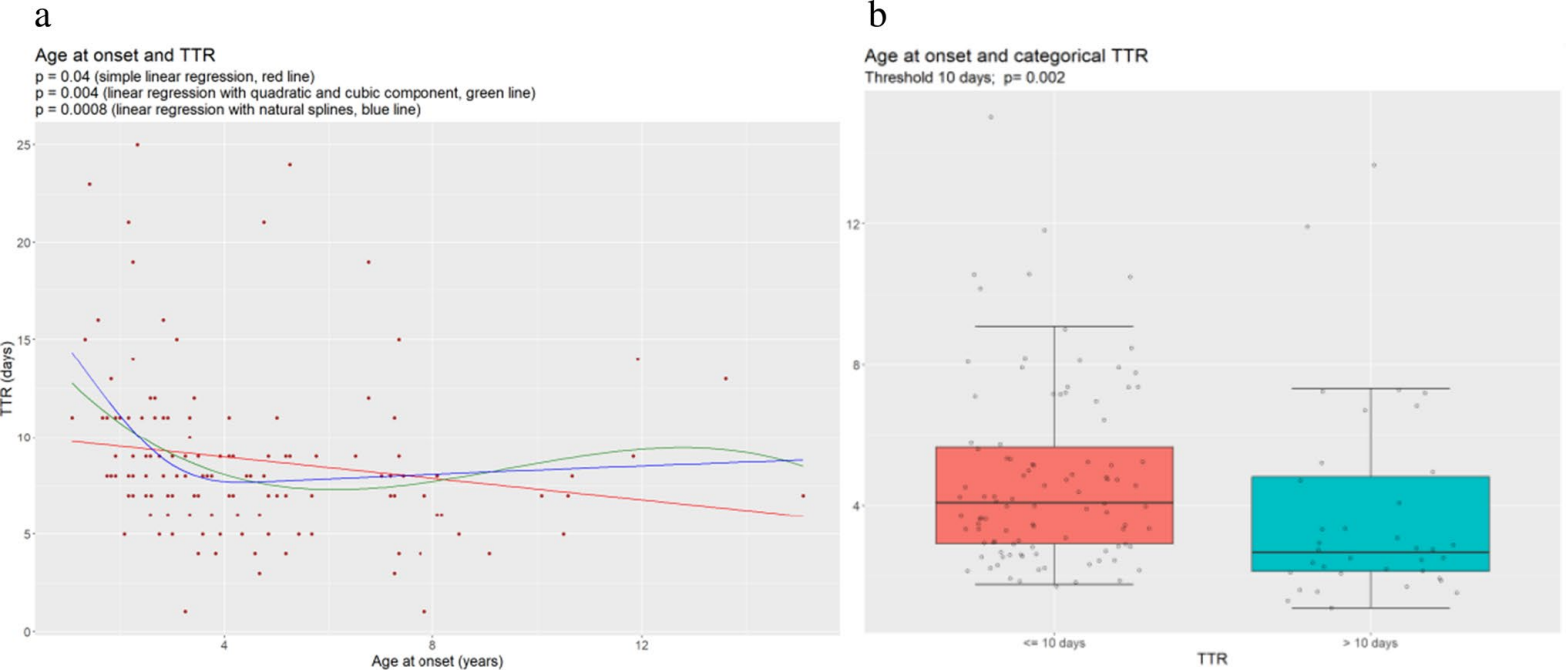
Correlation between age at onset and TTR (**a**) using TTR as a linear variable (**b**) using TTR as a categorical variable

**Fig. 2:**
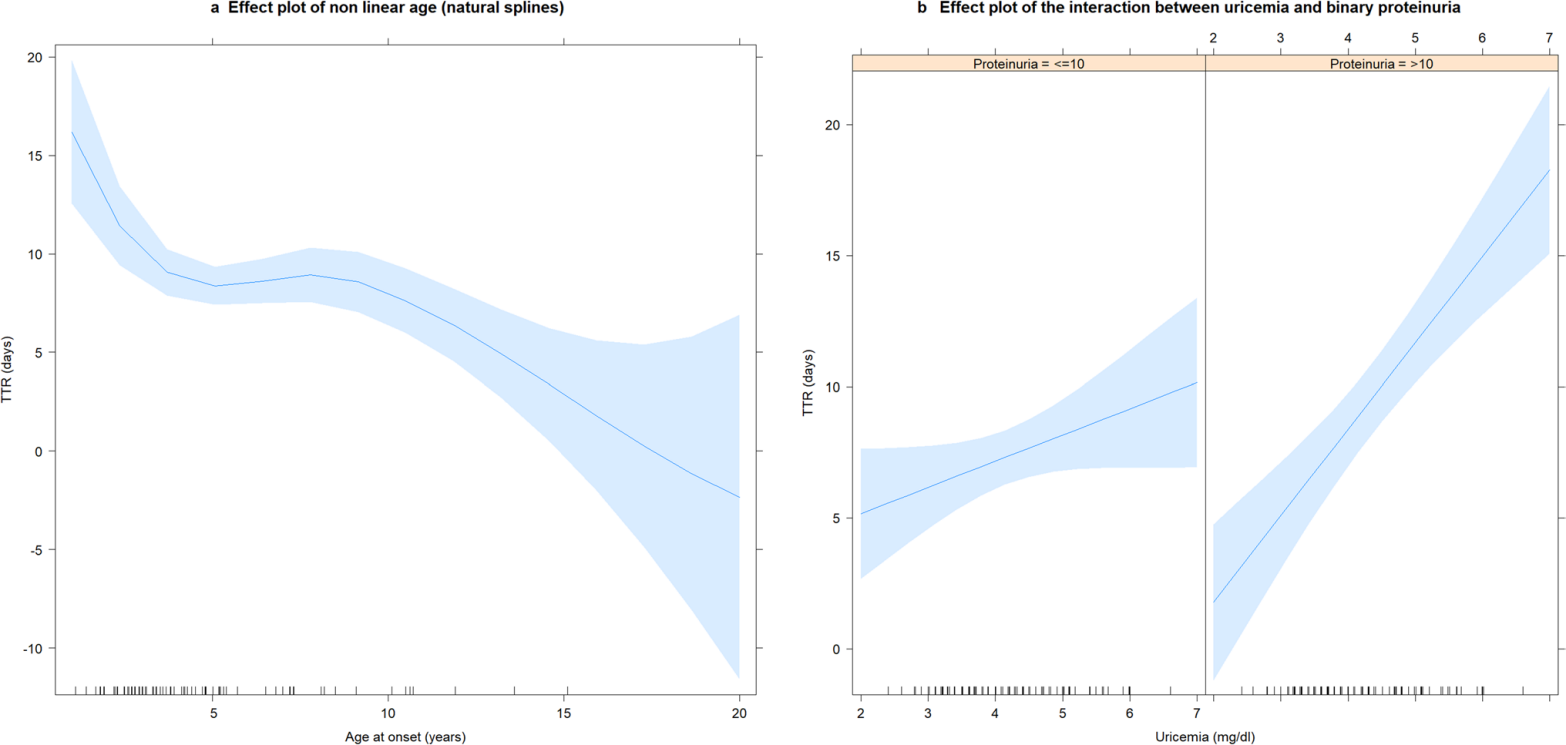
Effect plot of predictors for TTR in multiple regression model: **a** non linear age (natural splines) (**b**) interaction between uricemia and binary proteinuria

In simple linear regression models, TTR was directly correlated with some laboratory data at onset: serum uric acid (coefficient: 1.55; *p* = 0.0001), proteinuria (coefficient: 0.14; *p* = 0.0002), binary proteinuria (> = 10 mg/mg, coefficient: 2.6, *p* = 0.0005), serum potassium (coefficient: 1.9, *p* = 0.02), and urea (coefficient: 0.05; *p* = 0.05). Time to remission also showed an inverse correlation with age at onset (coefficient −0.27; *p* = 0.04) (Table 2). However, this relationship was not linear and it was better described by a polynomial regression including a quadratic and a cubic term (*p* = 0.004) or by a linear regression with natural splines (*p* = 0.0008), as shown in Fig. 1a. Time to remission became progressively shorter with age up to 4—5 years, it then remained stable, with some slight variation seen after 10 years of age. As a consequence, children with a TTR ≤ 10 days had a higher median age than children with a TTR > 10 days (4.1 vs 2.7 years, *p* = 0.002) (Fig. 1b). The best multiple linear regression model of TTR (both in terms of highest adjusted R squared and lowest model *p* value and AIC) included only hyperuricemia (*p* = 0.0000007), non-linear age (natural splines, *p* = 0.0006) and binary proteinuria (*p* = 0.02) and a positive interaction between binary proteinuria and uricemia (*p* = 0.004) as significant predictors of TTR. Therefore, TTR resulted positively related to hyperuricemia and proteinuria, and inversely related to age, especially in the first 4–5 years of life (Fig. 2). Moreover, because of the positive interaction between proteinuria and uricemia, TTR was significantly higher when the values of both variables increased (Fig. 2). When analyzing the correlation between age and those laboratory variables correlating with TTR, we observed that age was positively correlated with levels of uricemia only (coefficient: 0.07; p = 0.054; p = 0.005 with non linear age) and it was inversely correlated with proteinuria (coefficient: −1.4; p = 0.002). However, as shown by the multiple regression model, the effect of age on TTR is independent from the effects of uricemia and proteinuria (Fig. 2).

**Table 2:** TTR and variables at onset

Baseline variables	Univariate analysis		Multiple linear regression (final model) ***
	Coefficient estimate	*p* value	*p* value
Proteinuria (mg/mg)	0.13	**0.0002**	
Binary proteinuria (> = 10 mg/mg)	2.6	**0.0005**	**0.02**
Age at onset (years)	−0.27	**0.04**	
Age at onset (years, non linear, natural splines)		**0.0008**	**0.0006**
GFR (ml/min/1.73 m2)	−0.0045	0.36	
BUN (mg/dl)	0.05	0.053	
Serum total protein (g/dl)	0.02	0.96	
Serum albumin (g/dl)	0.26	0.72	
Serum K (mEq/l)	1.9	**0.01**	
Serum Na (mEq/l)	−0.15	0.14	
Urine output (ml/kg/h)	−0.804	0.16	
Uric acid (mg/dl)	1.55	**0.0001**	**0.0000007**
Hb (g/dl)	0.098	0.76	
Weight gain ratio (%) *	0.40	0.93	
Systolic BP (sDS)	0.13	0.72	
Diastolic BP (sDS)	0.43	0.34	
Systolic BP > 90th percentile		0.18	
Diastolic BP > 90th percentile		0.73	
Albumin administration at onset**		0.07	
Interaction between binary proteinuria and uricemia			**p = 0.004**

* (Weight at onset – weight at remission) / weight at remission × 100

** Albumin was administered at onset to 48.5% of patients. Mean TTR was slightly higher in the group of those who received albumin (9.6 vs 8.1), but the difference was not significant

*** All variables with *p* value < 0.10 at simple linear regression analysis were considered for multiple linear regression. The final model was chosen in terms of lowest AIC, and included only non linear age, binary proteinuria, uricemia, and a positive interaction between binary proteinuria and uricemia, as shown in the table. This model had the highest adjusted R squared (0.35), the lowest model *p* value (5.6e-08) and the lowest AIC

In summary, these results show that TTR tends to be higher in younger children (under 5 years of age) and in children who have high values of both proteinuria and uricemia. None of the other variables considered showed significant correlation with TTR.

## Discussion

In our analysis, hyperuricemia and proteinuria were found to directly correlate with TTR, while age was inversely correlated with a non-linear pattern, especially in the first years of life. The multiple regression analysis confirmed these associations, and showed a significant positive interaction between proteinuria and uricemia, so that each of these two variables is a much stronger predictor of TTR when the values of the other variable are higher.

Time to remission has been largely considered a potential predictive factor in childhood INS. Some authors have found a significant correlation between TTR and outcome [5–9], while other authors have not identified a prognostic role for TTR [10]. Conversely, to the best of our knowledge, TTR has never been thought of as a consequence of a different clinical status at onset. Therefore, in our post hoc analysis, we analyzed the possible influence of clinical and laboratory parameters at INS onset on prolonging TTR in children.

The degree of proteinuria was directly correlated with TTR (and showed a strong positive interaction with uricemia). There are many possible explanations for the role of proteinuria in determining TTR, but it seems rather intuitive that it is a fundamental expression of the severity of the disease at onset and certainly correlated to many other important clinical features (for example, proteinemia and water and sodium balance). Also uricemia in our model was a very strong independent predictor of TTR (interacting with proteinuria). The reasons for that are probably less obvious. We hypothesize that part of the explanation can be found in the relation between hyperuricemia and hypovolemia. Indeed, high level of serum uric acid in dehydration are reported in literature. Kuge et al. [12] supported this relationship in children with acute gastroenteritis. Roncal-Jimenez C et al. [13] proposed that hyperuricemia is one of the mechanisms by which chronic recurrent dehydration leads to chronic kidney disease. Nevertheless, we cannot exclude that other mechanisms are involved in uric acid elevation [14] in patients with nephrotic syndrome: for example, hyperuricemia could simply be an independent indicator of the severity of the disease at onset, and therefore of its ability to respond to therapy.

Hypovolemia in nephrotic syndrome has been largely studied as one of the two main mechanisms that cause nephrotic edema [15]. According to the “underfill” theory [15], hypovolemia is implicated in nephrotic edema pathogenesis. Therefore, volemia is one of the cardinal features to be evaluated in a patient with nephrotic syndrome, in order to better understand the pathogenesis of the edema and how to manage the patient properly [16]. Evaluating fluid status is often a challenge in these children, especially when the patient presents with INS onset and a concurrent acute gastroenteritis infection [17]. Kapur et al. [18] suggested that intravascular volume can be better estimated by calculating the fractional excretion of sodium (FeNa) in patients with INS. In particular, in their study, FeNa < 0.2% identified hypovolemic patients. Unfortunately, in our cohort urine sodium was not routinely checked, making FeNa calculation impossible. Further studies should include urinary sodium measurements among the variables to be evaluated at INS onset. Moreover, exploring whether patients with concurrent acute gastroenteritis at INS onset have a longer TTR, due to their hypovolemic status, would be interesting.

Finally, in our cohort, age was inversely and independently correlated with TTR in the first few years of life. Therefore, children under 5 years of age tend to show longer TTR. Sinha et al. [19] and Kabuki et al. [20] identified age at onset < 4 years as a risk factor for frequent relapses. Andersen et al. [21] reported that the ratio of frequently relapsing and steroid-dependent patients declined as age at onset increased, and nearly 90% of patients with age at onset < 4 years became frequent relapsers or steroid-dependent. Pasini et al. [11] also identified age as a predictive factor for both relapse and steroid-dependency. In our patients, the correlation between age and TTR may therefore play a confounding role in assessing TTR as a predictive factor, due to the prognostic impact of younger age. Age was directly correlated with hyperuricemia and inversely correlated with proteinuria. In children and adolescents, the reference values for uric acid increases gradually with age, with a difference between the sexes arising at about 12 years of age [22]. This could explain the association between uric acid levels and age in our cohort. To explain the inverse correlation between age and proteinuria, we hypothesize that, in children with a lower age at onset, less specific clinical signs could lead to a later diagnosis with higher levels of proteinuria, possibly leading to more pronounced hypovolemia and longer TTR in these patients. Moreover, while the reasons for that are not obvious, the different state of maturation and reactivity of the immune system in the first few years of life can certainly be part of an explanation. Indeed, the immunological involvement in the pathogenesis of SNI is well known, although multiple mechanisms have been hypothesized as the cause [23].

However, multiple regression confirms that uric acid level is by far the strongest predictor of TTR, even correcting for age and proteinuria. Therefore, hyperuricemia appears to be an independent predictor of longer TTR at all ages. Nonetheless, we cannot exclude that other mechanisms are involved in the longer TTR seen in younger children.

As a side note, we excluded seven patients with a TTR > 28 days from this analysis, due to their late response [3]. However, they showed no significantly worse biochemical parameters at onset compared to patients with a TTR > 10 days. Therefore, TTR in these patients may also be influenced by some other unknown factors involving their immunological response or for pharmacogenetic or pharmacodynamic reasons.

Further prospective studies are needed to confirm these results, possibly including the evaluation of additional hypovolemic parameters: urinary sodium for FeNa, weight gain assessed, if possible, even using bioelectrical impedance methods to estimate fluid excess [24].

## Conclusions

In our analysis, TTR appears to be an epiphenomenon of a more compromised clinical status at INS diagnosis, characterized by higher uricemia (possibly as a sign of more severe hypovolemia), higher proteinuria, or younger age. While we have discussed possible explanations for that behavior, additional mechanisms may certainly play a role in prolonging TTR. Further studies will be necessary to better characterize the relationship between volemic state at INS onset and response to therapy, especially in different age groups.

## Data Availability

No datasets were generated or analysed during the current study.

## References

[1] Vivarelli M, Gibson K, Sinha A, Boyer O (2023) Childhood nephrotic syndrome. Lancet 402:809–824. 10.1016/S0140-6736(23)01051-6 37659779 10.1016/S0140-6736(23)01051-6

[2] Pasini A, Aceto G, Ammenti A, Ardissino G et al (2015) Best practice guidelines for idiopathic nephrotic syndrome: recommendations versus reality. Pediatr Nephrol 30(1):91–101. 10.1007/s00467-014-2903-7 25127916 10.1007/s00467-014-2903-7 PMC4240913

[3] Rovin BH, Adler SG, Barratt J et al (2021) KDIGO 2021 Clinical practice guideline for the management of glomerular diseases. Kidney Int 100(4S):S1–S276. 10.1016/j.kint.2021.05.021 34556256 10.1016/j.kint.2021.05.021

[4] Trautmann A, Boyer O, Hodson E et al (2023) IPNA clinical practice recommendations for the diagnosis and management of children with steroid-sensitive nephrotic syndrome. Pediatr Nephrol 38(3):877–919. 10.1007/s00467-022-05739-3 36269406 10.1007/s00467-022-05739-3 PMC9589698

[5] Constantinescu AR, Shah HB, Foote EF, Weiss LS (2000) Predicting first-year relapses in children with nephrotic syndrome. Pediatrics 105(3):492–495. 10.1542/peds.105.3.492 10699098 10.1542/peds.105.3.492

[6] Yap HK, Han EJS, Heng CK, Gong WK (2001) Risk factors for steroid dependency in children with idiopathic nephrotic syndrome. Pediatr Nephrol 16:1049–1052. 10.1007/s004670100024 11793098 10.1007/s004670100024

[7] Letavernier B, Letavernier E, Leroy S, Baudet-Bonneville V, Bensman A, Ulinski T (2008) Prediction of high degree steroid dependency in pediatric idiopathic nephrotic syndrome. Pediatr Nephrol 23:2221–2226. 10.1007/s00467-008-0914-y 18618150 10.1007/s00467-008-0914-y

[8] Prasun B, Payas J, Sujaya M (2017) Prediction of relapses in children with idiopathic steroid sensitive nephrotic syndrome: a retrospective study. Int J Contemp Pediatr 4(1):57–61. 10.18203/2349-3291.ijcp20164437

[9] Vivarelli M, Moscaritolo E, Tsalkidis A, Massella L, Emma F (2010) Time for initial response to steroids is a major prognostic factor in idiopathic nephrotic syndrome. J Pediatr 156:965–971. 10.1016/j.jpeds.2009.12.020 20223477 10.1016/j.jpeds.2009.12.020

[10] Dossier C, Delbet JD, Boyer O et al (2019) Five-year outcome of children with idiopathic nephrotic syndrome: the NEPHROVIR population-based cohort study. Pediatr Nephrol 34(4):671–678. 10.1007/s00467-018-4149-2 30552564 10.1007/s00467-018-4149-2

[11] Pasini A, Bertulli C, Casadio L, Corrado C et al (2021) Childhood idiopathic nephrotic syndrome: does the initial steroid treatment modify the outcome? a multicentre prospective cohort study. Front Pediatr 9:627636. 10.3389/fped.2021.627636 34307246 10.3389/fped.2021.627636 PMC8295604

[12] Kuge R, Morikawa Y, Hasegawa Y (2017) Uric acid and dehydration in children with gastroenteritis. Pediatr Int 59(11):1151–1156. 10.1111/ped.13366 28714223 10.1111/ped.13366

[13] Roncal-Jimenez C, Lanaspa MA, Jensen T et al (2015) Mechanisms by which dehydration may lead to chronic kidney disease. Ann Nutr Metab 66 Suppl 3:10–3. 10.1159/000381239 10.1159/000381239 26088040

[14] Du L, Zong Y, Li H et al (2024) Hyperuricemia and its related diseases: mechanisms and advances in therapy. Signal Transduct Target Ther 9(1):212. 10.1038/s41392-024-01916-y 39191722 10.1038/s41392-024-01916-y PMC11350024

[15] Pasini A, Benetti E, Conti G, Ghio L et al (2017) The Italian Society for Pediatric Nephrology (SINePe) consensus document on the management of nephrotic syndrome in children: Part I - Diagnosis and treatment of the first episode and the first relapse. Ital J Pediatr 43(1):41. 10.1186/s13052-017-0356-x 28427453 10.1186/s13052-017-0356-x PMC5399429

[16] McCaffrey J, Lennon R, Webb NJA (2016) The non-immunosuppressive management of childhood nephrotic syndrome. Pediatr Nephrol 31(9):1383–1402. 10.1007/s00467-015-3241-0 26556028 10.1007/s00467-015-3241-0 PMC4943972

[17] Marzuillo P, Guarino S, Apicella A et al (2017) Assessment of volume status and appropriate fluid replenishment in the setting of nephrotic syndrome. J Emerg Med 52(4):e149–e152. 10.1016/j.jemermed.2016.07.089 28209267 10.1016/j.jemermed.2016.07.089

[18] Kapur G, Valentini RP, Imam AA, Mattoo TK (2009) Treatment of severe edema in children with nephrotic syndrome with diuretics alone - A prospective study. Clin J Am Soc Nephrol 4(5):907–913. 10.2215/CJN.04390808 19406963 10.2215/CJN.04390808 PMC2676186

[19] Sinha A, Hari P, Sharma PK, Gulati A et al (2012) Disease course in steroid sensitive nephrotic syndrome. Indian Pediatr 49(11):881–887. 10.1007/s13312-012-0220-4 22791676 10.1007/s13312-012-0220-4

[20] Kabuki N, Okugawa T, Hayakawa H, Tomizawa S, Kasahara T, Uchiyama M (1998) Influence of age at onset on the outcome of steroid-sensitive nephrotic syndrome. Pediatr Nephrol 12(6):467–470. 10.1007/s004670050489 9745870 10.1007/s004670050489

[21] Andersen RF, Thrane N, Noergaard K, Rytter L, Jespersen B, Rittig S (2010) Early age at debut is a predictor of steroid-dependent and frequent relapsing nephrotic syndrome. Pediatr Nephrol 25:1299–1304. 10.1007/s00467-010-1537-7 20446093 10.1007/s00467-010-1537-7

[22] Kubota M (2019) Hyperuricemia in children and adolescents: present knowledge and future directions. J Nutr. Metab. 3480718:3480718. 10.1155/2019/3480718 10.1155/2019/3480718 PMC6525889 31192008

[23] Colucci M, Corpetti G, Emma F, Vivarelli M (2018) Immunology of idiopathic nephrotic syndrome. Pediatr Nephrol 33(4):573–584. 10.1007/s00467-017-3677-5 28451893 10.1007/s00467-017-3677-5

[24] Bozzetto S, Piccoli A, Montini G (2010) Bioelectrical impedance vector analysis to evaluate relative hydration status. Pediatr Nephrol 2:329–334. 10.1007/s00467-009-1326-3 10.1007/s00467-009-1326-3 19876654

